# Investigation of Nutrition Knowledge Levels Among Fitness Center Members According to Their Metabolic Profiles

**DOI:** 10.3390/metabo16100796

**Published:** 2026-10-21

**Authors:** Murat Bekleyiş Apaydın, Rıdvan Ergin

**Affiliations:** 1 Department of Coaching Education, Faculty of Sport Sciences, Niğde Ömer Halisdemir University, Niğde 51240, Türkiye; mapaydin@ohu.edu.tr; 2 Department of Physical Education and Sports, Faculty of Sport Sciences, Recep Tayyip Erdoğan University, Rize 53100, Türkiye

**Keywords:** cardiometabolic health, fitness, metabolic profile indicators, nutrition knowledge level, physical activity

## Abstract

**Background**: This research aimed to examine the associations between the total “Nutrition Knowledge Scale” score and metabolic profile indicators among fitness center members, and to explore whether selected nutrition knowledge items differed according to daily meal consumption. **Methods**: The research, conducted using a cross-sectional and relational screening model, included a total of 230 volunteers, consisting of 67 women and 163 men. The participants’ HDL, LDL, fasting glucose, and triglyceride levels were obtained from biochemical analysis results via the e-Nabız system, while their nutrition knowledge levels were assessed using the “Nutrition Knowledge Scale”. Descriptive statistics, simple and multiple linear regression analyses, and one-way ANOVA were used for data analysis. **Results**: The total “Nutrition Knowledge Scale” score was significantly associated with LDL levels after adjustment for BMI and gender (β = −0.136, *p* = 0.037), whereas no statistically significant associations were observed with HDL, fasting glucose, or triglyceride levels. In secondary exploratory analyses, selected nutrition knowledge items showed significant associations with LDL levels, while the vitamin B12-related item was positively but not significantly associated with triglyceride levels (β = 0.099, *p* = 0.136). Additionally, responses to three selected nutrition knowledge items differed significantly according to daily meal consumption, with small effect sizes (η^2^ = 0.029–0.046). **Conclusions**: Overall nutrition knowledge was associated with LDL levels after adjustment for BMI and gender, but not with HDL, fasting glucose, or triglyceride levels. Secondary exploratory findings indicated associations between selected nutrition knowledge items, LDL levels, and daily meal consumption. Causal inference is limited by the cross-sectional design.

## 1. Introduction

In recent years, personalized nutrition approaches based on individuals’ metabolic profiles are becoming increasingly important for improving health outcomes and physical performance. Differences among people in genetics, eating habits, lifestyle, and metabolic responses lead to varying reactions to the same nutrition approach and make it necessary to develop personalized nutrition strategies [1,2]. The metabolic profile is defined as a biochemical indicator that reflects the quantitative and qualitative composition of metabolites found in a person’s blood, urine, or other biological samples, showing the combined effects of genetic makeup, nutrition, lifestyle, environmental factors, and physiological state. This profile is used as an important tool for identifying metabolic differences between individuals and developing personalized nutrition strategies [3,4]. In this research, the concept of metabolic profile was evaluated through routine biochemical indicators such as individuals’ HDL (High-Density Lipoprotein), LDL (Low-Density Lipoprotein), fasting glucose, and triglyceride levels. These indicators are among the key biochemical parameters used to assess individuals’ cardiometabolic health [5,6]. Also, differences between individuals in terms of energy expenditure, substrate use (carbohydrate and fat oxidation), and metabolic flexibility play an important role in shaping metabolic traits [7]. In addition to that, it has been shown that metabotype-based personalized nutrition interventions can positively influence individuals’ diet quality and metabolic health parameters [8,9].

In this context, it is thought that for personalized nutrition approaches based on metabolic traits to be effectively applied, it depends not only on identifying individuals’ biological differences but also on assessing their level of nutrition knowledge, awareness, and ability to apply this information in their daily lives.

Understanding the relationship between nutrition habits, energy balance, and metabolic health in individuals with high physical activity levels is important for supporting performance and maintaining healthy weight management [10]. Studies have shown that people with high nutrition literacy and health awareness are more likely to adopt healthy eating behaviors that reduce the risk of metabolic syndrome [11]. Prospective studies suggest that increasing physical activity levels is linked to adopting a healthy lifestyle and may help lower the risk of developing metabolic syndrome [12]. Research on fitness center members and physically active people shows that nutrition knowledge is linked to healthy eating habits, but just being physically active does not guarantee enough nutrition knowledge on its own [13,14]. Similarly, among individuals who received sports training, nutrition education was associated with higher levels of nutrition knowledge [15]. Among fitness-center members, nutrition and exercise habits were found to vary according to factors such as age, educational level, and weekly exercise frequency [16]. In addition, nutrition education was shown to significantly improve nutrition knowledge among sports high school students, emphasizing the importance of individualized nutrition guidance [17]. Also, it is reported that healthy eating habits are influenced not just by biological risk perception, but by multidimensional factors like personal motivation, psychological state, and social environment [18,19].

The aim of this research was to examine the associations between the total Nutrition Knowledge Scale score and metabolic profile indicators (HDL, LDL, fasting glucose, and triglycerides) among fitness center members, while accounting for body mass index (BMI) and gender. In addition, selected nutrition knowledge items were examined as secondary exploratory analyses to determine whether individual item responses were associated with metabolic profile indicators and differed according to daily meal consumption. The hypotheses developed for this purpose were as follows:

**H1.**  *The total “Nutrition Knowledge Scale” score is significantly associated with metabolic profile indicators (HDL, LDL, fasting glucose, and triglycerides) among fitness center members.*

**H2.**  *Responses to selected nutrition knowledge scale items differ significantly according to fitness center members’ daily meal consumption groups.*

## 2. Materials and Methods

### 2.1. Research Model

This research is a quantitative study conducted in a cross-sectional and relational survey design to examine the relationship between fitness center members’ metabolic profile indicators and their nutrition knowledge levels. The research evaluated the relationships between participants’ metabolic profile indicators (HDL, LDL, fasting glucose, and triglycerides), their nutrition knowledge levels, and their meal consumption habits.

### 2.2. Study Group

The study group initially consisted of 231 participants (163 men and 68 women) aged 18 years and over who had been regularly performing CrossFit or strength exercises at fitness centers at least three days per week for the past two years and volunteered to participate in the research. One participant was excluded from the statistical analyses due to an implausible fasting glucose value (1 mg/dL). Therefore, the final analytical sample consisted of 230 participants (163 men and 67 women).

An a priori power analysis was conducted using G*Power 3.1.9.7. Based on a previously reported correlation coefficient of r = 0.25 [20], a two-tailed analysis with α = 0.05 and 95% power indicated a minimum required sample size of 202 participants. The final analytical sample of 230 participants exceeded this requirement.

The criteria for inclusion in the research were determined as follows:

- Be 18 or older and willing to participate in the research;
- Regularly going to the fitness center;
- Having HDL, LDL, fasting glucose, and triglyceride laboratory results recorded in the e-Nabız system (Personal Health Record System) within the preceding six months and consenting to share these results with the researchers.

### 2.3. Data Collection Tools

#### 2.3.1. Personal Information Form

The personal information form prepared by the researchers includes participants’ age, gender, education level, income level, marital status, daily meal count, height [centimeters (cm)], and body weight [kilograms (kg)]. Participants’ BMI was calculated by dividing body weight (kg) by the square of height [m (meters)^2^] (BMI = kg/m^2^) [21].

#### 2.3.2. Metabolic Profile Indicators

Participants’ HDL, LDL, fasting glucose, and triglyceride levels were obtained from laboratory test results recorded in the e-Nabız system. These metabolic indicators were measured in the fasting state and were treated as continuous variables in the analyses, with no categorical classification applied.

#### 2.3.3. Nutrition Knowledge Scale

The research used the “Nutrition Knowledge Scale” developed by Yılmaz et al. [22], which has been validated and tested for reliability in Turkish. The scale consists of 31 items rated on a five-point Likert scale, with response options scored from 4 (“Strongly agree”) to 0 (“Strongly disagree”). Before statistical analysis, the ten negatively worded items (1, 5, 6, 9, 10, 16, 17, 21, 27, and 28) were reverse-scored in accordance with the scale’s scoring procedure. Both the total scale score and the item-level analyses were based on the appropriately scored values. Higher total scores indicate greater nutrition knowledge.

In this research, the scale’s Cronbach’s alpha internal consistency coefficient was calculated as α = 0.797 and was found to be at an acceptable level.

For the secondary exploratory analyses, selected items from the Nutrition Knowledge Scale were included based on their relevance to nutrients and dietary components considered important for nutrition and metabolic health among fitness center members. These items addressed omega-3 fatty acids, calcium, vitamin D, vitamin B12, carbohydrates, sugars, and cholesterol. The selected items were therefore examined individually to explore their associations with metabolic profile indicators and daily meal consumption patterns. These analyses were considered secondary and exploratory.

### 2.4. Data Analysis

The Statistical Package for the Social Sciences (SPSS) version 29.0 was used for all statistical analyses. The suitability of the data for a normal distribution was evaluated by examining the skewness and kurtosis coefficients, and variables within the ±1.5 limits were considered to show a normal distribution [23]. Descriptive statistics are presented as the number of participants (*n*), mean ($\bar{x}$), and standard deviation (SD).

The total “Nutrition Knowledge Scale” score was used as the primary nutrition knowledge measure. Multiple linear regression analyses were performed to examine the associations between the total “Nutrition Knowledge Scale” score and HDL, LDL, fasting glucose, and triglyceride levels, with BMI and gender included as covariates. Selected nutrition knowledge items were additionally examined using simple linear regression analyses as secondary exploratory analyses. One-way analysis of variance (ANOVA) was used to compare responses to selected nutrition knowledge items according to daily meal consumption groups. The Tukey HSD test was used for post hoc pairwise comparisons when the homogeneity of variances assumption was met, whereas the Games–Howell test was used when this assumption was not met. Effect sizes for the ANOVA results were evaluated using the eta-squared (*η*^2^) coefficient. For eta squared values, 0.01 was considered small, 0.06 medium, and 0.14 large effect size thresholds [24]. The level of statistical significance was accepted as *p* < 0.05.

## 3. Results

Looking at Table 1, it can be seen that 71.7% of the participants (*n* = 165) are between 18 and 28 years old, 17.8% (*n* = 41) are between 29 and 39 years old, and 10.4% (*n* = 24) are 40 years old and above. 70.9% of the participants (*n* = 163) are male, and 29.1% (*n* = 67) are female. In terms of education, 69.1% of the participants (*n* = 159) have a bachelor’s degree, 23.5% (*n* = 54) have a high school diploma, and 7.4% (*n* = 17) have postgraduate education. Regarding income, 41.7% of the participants (*n* = 96) have a monthly income of 32,001 TL and above, 38.7% (*n* = 89) have 28,000 TL and below, and 19.6% (*n* = 45) have between 28,001 and 32,000 TL. Regarding daily meal consumption, 50.9% (*n* = 117) of the participants consumed 1–2 meals, 43.9% (*n* = 101) consumed 3–4 meals, and 5.2% (*n* = 12) consumed four or more meals. In terms of marital status, 77% of the participants (*n* = 177) are single, 19.6% (*n* = 45) are married, and 3.5% (*n* = 8) are divorced.

In Table 2, the participants’ mean height was 174.7 ± 8.9 cm, body weight 75.9 ± 15.1 kg, and BMI 24.7 ± 3.8 kg/m^2^. Looking at the metabolic profile indicators, the average HDL level was 60.1 ± 18.1 mg/dL, LDL level 76.6 ± 27.3 mg/dL, fasting glucose level 97.4 ± 14.2 mg/dL, and triglyceride level 83.1 ± 36.1 mg/dL. The mean total nutrition knowledge score of the participants was 66.1 ± 15.7 (range: 19–97).

In the initial simple linear regression analyses, the total “Nutrition Knowledge Scale” score was significantly associated with LDL levels (β = −0.154, R^2^ = 0.024, F = 5.505, t = −2.346, *p* = 0.020), whereas no statistically significant associations were observed with HDL (β = 0.052, R^2^ = 0.003, F = 0.612, t = 0.782, *p* = 0.435), fasting glucose (β = 0.022, R^2^ < 0.001, F = 0.108, t = 0.328, *p* = 0.743), or triglyceride levels (β = −0.015, R^2^ < 0.001, F = 0.048, t = −0.220, *p* = 0.826).

To account for the potential influence of BMI and gender, multiple linear regression analyses were subsequently conducted. As shown in Table 3, the total “Nutrition Knowledge Scale” score remained significantly associated with LDL levels after adjustment for BMI and gender (β = −0.136, R^2^ = 0.062, F = 4.943, t = −2.099, *p* = 0.037). No statistically significant association was observed between the total “Nutrition Knowledge Scale” score and HDL, fasting glucose, or triglyceride levels.

Table 4 presents the results of the secondary exploratory simple linear regression analyses examining associations between selected “Nutrition Knowledge Scale” items and metabolic profile indicators. Among the selected items, a statistically significant association was observed between responses to item 16 (“Sugars and sugary foods should be consumed as much as possible”) and LDL levels (β = 0.168, R^2^ = 0.028, F = 6.601, t = 2.569, *p* = 0.011). Responses to item 18 (“Is vitamin B12 deficiency the cause of diseases (nervous system disorders and anemia)?”) showed a positive but statistically non-significant association with triglyceride levels (β = 0.099, R^2^ = 0.010, F = 2.235, t = 1.495, *p* = 0.136). Similarly, responses to item 29 (“Calcium and vitamin D are important for health.”) were not significantly associated with fasting glucose levels (β = −0.035, R^2^ = 0.001, F = 0.276, t = −0.525, *p* = 0.600). Among the associations presented in Table 4, only the association between item 16 and LDL levels reached statistical significance.

As shown in Table 5, significant associations were observed between responses to selected nutrition knowledge items and LDL levels, whereas no statistically significant associations were found with fasting glucose levels. Responses to item 4 (“Fish is the best source of omega-3.”) were significantly and negatively associated with LDL levels (β = −0.185, R^2^ = 0.034, F = 8.042, t = −2.836, and *p* = 0.005), whereas the association with fasting glucose levels was not statistically significant (β = −0.121, R^2^ = 0.015, F = 3.408, t = −1.846, and *p* = 0.066). Similarly, responses to item 7 (“Milk and dairy products are the best sources of calcium.”) were significantly and negatively associated with LDL levels (β = −0.201, R^2^ = 0.040, F = 9.579, t = −3.095, and *p* = 0.002), but not with fasting glucose levels (β = −0.072, R^2^ = 0.005, F = 1.177, t = −1.085, and *p* = 0.279). Responses to item 13 (“Bread, pasta, rice, and bulgur, which are rich in carbohydrates, are foods in the grain group.”) also showed a significant negative association with LDL levels (β = −0.257, R^2^ = 0.066, F = 16.146, t = −4.018, and *p* < 0.001), whereas the association with fasting glucose levels was not statistically significant (β = −0.064, R^2^ = 0.004, F = 0.932, t = −0.965, and *p* = 0.335).

As shown in Table 6, responses to three selected nutrition knowledge items differed significantly across daily meal consumption groups.

Responses to item 4 (“Fish is the best source of omega-3.”) differed significantly across daily meal consumption groups (F = 4.860, *p* = 0.009, η^2^ = 0.041). Tukey’s post hoc analysis indicated significant differences between individuals consuming 1–2 meals and those consuming 3–4 meals, as well as between individuals consuming 1–2 meals and those consuming four or more meals. The mean score was higher among individuals consuming 1–2 meals (2.50 ± 1.45) than among those consuming 3–4 meals (2.01 ± 1.53) or four or more meals (1.42 ± 1.51).

For item 6 (“Cholesterol is only found in plant foods.”), a significant difference was also observed across daily meal consumption groups (F = 3.412, *p* = 0.035, η^2^ = 0.029). Because the homogeneity of variance assumption was not met, the Games–Howell post hoc test was used. The significant difference was observed between the 1–2 meal and 3–4 meal groups, with mean scores of 1.64 ± 1.22 and 2.06 ± 1.32, respectively.

Responses to item 8 (“Vitamin D can be produced by the body through sunlight.”) also differed significantly across daily meal consumption groups (F = 5.429, *p* = 0.005, η^2^ = 0.046). Tukey’s post hoc analysis showed a significant difference between the 1–2 meal and four-or-more-meal groups. The mean score was higher in the 1–2 meal group (2.50 ± 1.38) than in the four-or-more-meal group (1.25 ± 1.55).

The effect sizes for items 4, 6, and 8 were η^2^ = 0.041, η^2^ = 0.029, and η^2^ = 0.046, respectively, indicating small effects of daily meal consumption group on responses to these nutrition knowledge items.

## 4. Discussion

This research aimed to examine the associations between nutrition knowledge and metabolic profile indicators among individuals who regularly attend fitness centers, with the total “Nutrition Knowledge Scale” score used as the primary measure of nutrition knowledge. The findings showed that the total “Nutrition Knowledge Scale” score was significantly associated with LDL levels after adjustment for BMI and gender, whereas no statistically significant associations were observed with HDL, fasting glucose, or triglyceride levels. In addition, selected nutrition knowledge items were examined as secondary exploratory analyses, and some of these items showed statistically significant associations with LDL levels or differences according to daily meal consumption groups. Overall, these findings suggest that the relationship between nutrition knowledge and metabolic profile indicators may vary across different aspects of nutrition knowledge among physically active individuals.

B vitamins play important roles in energy metabolism and various cellular processes [25], while vitamin B12 is essential for DNA synthesis, red blood cell formation, and normal nervous system function [26]. Karacaer and Tuncer [27] reported an association between vitamin B12 levels and some metabolic parameters in individuals with type 2 diabetes. In the present research, the selected item concerning vitamin B12 deficiency showed a positive but statistically non-significant association with triglyceride levels (β = 0.099, R^2^ = 0.010, *p* = 0.136). Given that this analysis was part of the secondary exploratory analyses, the observed association should be interpreted cautiously and does not provide sufficient evidence of a statistically significant relationship between nutrition knowledge concerning vitamin B12 deficiency and triglyceride levels.

In the present research, responses to item 4 (“Fish is the best source of omega-3.”) were significantly and negatively associated with LDL levels (β = −0.185, R^2^ = 0.034, *p* = 0.005), whereas no statistically significant association was observed with fasting glucose levels (*p* = 0.066). Omega-3 fatty acids are biologically active components that play important roles in lipid metabolism and cardiometabolic health [28]. The observed association between the nutrition knowledge item concerning dietary sources of omega-3 fatty acids and LDL levels suggests that this specific aspect of nutrition knowledge was statistically related to a lipid-related metabolic indicator in the present sample. However, the relatively small explained variance (R^2^ = 0.034, corresponding to 3.4% of the variance) indicates that this item accounted for only a limited proportion of the variation in LDL levels. Moreover, because this was a secondary exploratory analysis based on a cross-sectional research design, the observed association should not be interpreted as evidence of a causal relationship.

Nutrition plays an important role in maintaining bone health throughout life [29]. Calcium is a major mineral component of bone and tooth tissue and also contributes to muscle function, nerve transmission, and cellular processes [30]. Adequate calcium intake is therefore particularly relevant for physically active individuals in maintaining bone health. In the present research, responses to item 7 (“Milk and dairy products are the best sources of calcium.”) were significantly and negatively associated with LDL levels (β = −0.201, R^2^ = 0.040, *p* = 0.002), whereas no statistically significant association was observed with fasting glucose levels (*p* = 0.279). Adequate calcium intake and appropriate resistance exercise are considered important for maintaining bone health in physically active individuals [31,32]. However, the present finding represents an association between responses to a specific nutrition knowledge item and LDL levels and should not be interpreted as evidence that knowledge about calcium sources directly affects LDL levels. The relatively small explained variance (R^2^ = 0.040) also indicates that this item accounted for a limited proportion of the variation in LDL levels.

Among dietary factors, high consumption of free sugars has been associated with adverse effects on energy balance, insulin responses, and lipid metabolism [33]. In the present research, responses to item 16 (“Sugars and sugary foods should be consumed as much as possible”) were positively and significantly associated with LDL levels (β = 0.168, R^2^ = 0.028, *p* = 0.011). This finding indicates that responses to this specific nutrition knowledge item were statistically associated with LDL levels in the present sample. However, the relatively small explained variance (R^2^ = 0.028) indicates that the item accounted for only a limited proportion of the variation in LDL levels. In addition, the wording of the item reflects a nutrition knowledge statement rather than a direct measure of actual sugar consumption; therefore, the finding should not be interpreted as evidence that sugar consumption itself caused higher LDL levels. Moreover, during exercise, processes like the regulation of glucose metabolism, hormonal responses, and liver glucose production come into play due to the increased energy demand [34]. As this was a secondary exploratory analysis within a cross-sectional study, causal interpretations are not warranted.

In the present research, responses to items 4, 6, and 8 differed significantly across daily meal consumption groups (*p* < 0.05). Meal patterns are an important lifestyle factor that may influence total energy intake, metabolic responses, and eating behaviors [35]. Çevik et al. [36] reported that nutrition-related attitudes among individuals who engage in fitness activities may vary according to certain demographic characteristics and that education and income levels may influence the nutrition knowledge dimension. In the present research, the observed differences in selected nutrition knowledge items across meal consumption groups suggest that nutrition knowledge and eating patterns may be related. However, these findings should be interpreted cautiously because the four-or-more-meal group included only 12 participants, which may limit the stability and generalizability of comparisons involving this group. In addition, the cross-sectional design does not allow the direction of the relationship between meal consumption patterns and nutrition knowledge to be established.

An important finding of the present research was the significant association between the total “Nutrition Knowledge Scale” score and LDL levels after adjustment for BMI and gender (β = −0.136, R^2^ = 0.062, t = −2.099, and *p* = 0.037). In contrast, no statistically significant associations were observed between the total nutrition knowledge score and HDL, fasting glucose, or triglyceride levels. These findings indicate that the relationship between overall nutrition knowledge and metabolic profile indicators was not consistent across all measured biomarkers. The persistence of the association with LDL after adjustment for BMI and gender suggests that the observed relationship was not fully explained by these two covariates. Nevertheless, the cross-sectional design of the research precludes causal inference, and the relatively modest explained variance indicates that factors beyond nutrition knowledge also contribute to variation in LDL levels.

## 5. Conclusions

This research examined the associations between nutrition knowledge and metabolic profile indicators among individuals who regularly attend fitness centers, using the total Nutrition Knowledge Scale score as the primary measure of nutrition knowledge. The mean HDL, LDL, fasting glucose, and triglyceride levels of the participants were within the respective clinical reference ranges.

The total Nutrition Knowledge Scale score was significantly associated with LDL levels after adjustment for BMI and gender, whereas no statistically significant associations were observed with HDL, fasting glucose, or triglyceride levels. In addition, secondary exploratory analyses of selected nutrition knowledge items identified significant associations between some items and LDL levels, while the association between the vitamin B12-related item and triglyceride levels was not statistically significant.

Furthermore, responses to three selected nutrition knowledge items differed significantly according to daily meal consumption groups, although the corresponding effect sizes were small. These findings suggest that specific aspects of nutrition knowledge may be related to metabolic profile indicators and meal consumption patterns among physically active fitness center members.

Overall, the findings indicate that nutrition knowledge may be relevant to metabolic health assessment in physically active individuals. However, these associations should be interpreted cautiously because of the cross-sectional design and the exploratory nature of the selected-item analyses. The findings do not establish causal relationships between nutrition knowledge, meal consumption patterns, and metabolic profile indicators.

### 5.1. Suggestions

According to the research findings, we have the following:

- Implementing assessment and education programs at fitness centers regarding individuals’ metabolic health and nutrition knowledge levels;
- Conducting more comprehensive studies that evaluate nutrition knowledge, food consumption habits, and metabolic indicators together in physically active individuals;
- In future studies, examining the relationship between nutrition knowledge and metabolic parameters using larger sample groups and long-term follow-up methods;
- It is suggested to increase collaboration between sports experts and nutrition professionals to develop personalized nutrition approaches for fitness center members.

### 5.2. Limitations

This research has several limitations. First, its cross-sectional design does not allow causal relationships to be established between nutrition knowledge, meal consumption patterns, and metabolic profile indicators. Second, although the total Nutrition Knowledge Scale score was used as the primary measure of nutrition knowledge, the analyses of individual scale items were secondary exploratory analyses and should therefore be interpreted cautiously (as multiple testing may increase the risk of Type I errors). Third, the four-or-more-meal group included only 12 participants, which may limit the stability and generalizability of comparisons involving this group. Finally, the research included BMI and gender as covariates in the multiple regression models; other potentially relevant factors may also influence metabolic profile indicators and were not accounted for in the present analyses.

## Figures and Tables

**Table 1:** Participants’ demographic characteristics.

Variables	Parameters	*n*	%
Age (years)	18–28	165	71.7
	29–39	41	17.8
	≥40	24	10.4
Gender	Male	163	70.9
	Female	67	29.1
Education Level	High school	54	23.5
	Bachelor’s	159	69.1
	Graduate	17	7.4
Income Status (TL)	≤28,000	89	38.7
	28,001–32,000	45	19.6
	≥32,001	96	41.7
Meal Consumption	1–2 meals	117	50.9
	3–4 meals	101	43.9
	≥4 meals	12	5.2
Marital Status	Married	45	19.6
	Single	177	77
	Divorced	8	3.5

TL: Turkish lira.

**Table 2:** Descriptive statistics on participants’ anthropometric measurements and metabolic profile indicators.

Variables	$\bar{x}$ ± sd
Height (cm)	174.7 ± 8.9
Body weight (kg)	75.9 ± 15.1
BMI (kg/m^2^)	24.7 ± 3.8
HDL (mg/dL)	60.1 ± 18.1
LDL (mg/dL)	76.6 ± 27.3
Fasting glucose (mg/dL)	97.4 ± 14.2
Triglyceride (mg/dL)	83.1 ± 36.1
Total Nutrition Knowledge Score	66.1 ± 15.7

mg/dL: milligram/deciliter.

**Table 3:** Multiple linear regression analysis of total nutrition knowledge score, BMI, and gender with metabolic profile indicators.

Variables	Predictor	β	95% CI for B	R^2^	F	t	*p*
HDL (mg/dL)	Total Score	0.058	−0.078 to 0.213	0.081	6.638	0.912	0.363
	BMI (kg/m^2^)	0.290	0.756 to 2.028			4.315	**<0.001**
	Gender	−0.039	−6.827 to 3.723			−0.580	0.563
LDL (mg/dL)	Total Score	−0.136	−0.457 to −0.014	0.062	4.943	−2.099	**0.037**
	Gender	0.195	3.644 to 19.720			2.864	**0.005**
	BMI (kg/m^2^)	0.003	−0.948 to 0.989			0.041	0.967
Fasting glucose (mg/dL)	Total Score	0.033	−0.082 to 0.142	0.115	9.762	0.529	0.597
	BMI (kg/m^2^)	0.339	0.793 to 1.774			5.155	**<0.001**
	Gender	−0.004	−4.202 to 3.941			−0.063	0.950
Triglycerides (mg/dL)	Total Score	0.007	−0.233 to 0.266	0.316	34.747	0.131	0.896
	BMI (kg/m^2^)	0.553	4.207 to 6.392			9.557	**<0.001**
	Gender	0.026	−7.006 to 11.131			0.448	0.654

β: Standardized regression coefficient; B: Unstandardized regression coefficient; 95% CI: 95% confidence interval for B; R^2^: Explained variance; F: F value for the regression model; Gender was coded as 1 = female and 2 = male. Multiple linear regression models included total “Nutrition Knowledge Scale” score, BMI and gender as predictors.

**Table 4:** Secondary exploratory regression analyses of selected nutrition knowledge items and metabolic profile indicators.

Scale Item	Metabolic Variables	$\bar{x}$ ± sd	β	95% CI for B	R^2^	F	t	*p*
18	Triglyceride (mg/dL)	83.1 ± 36.1	0.099	−0.001 to 0.007	0.010	2.235	1.495	0.136
16	LDL (mg/dL)	76.6 ± 27.3	0.168	0.002 to 0.017	0.028	6.601	2.569	**0.011**
29	Fasting glucose (mg/dL)	97.4 ± 14.2	−0.035	−0.018 to 0.011	0.001	0.276	−0.525	0.600

β: Standardized regression coefficient; B: Unstandardized regression coefficient; 95% CI: 95% confidence interval for B.

**Table 5:** Secondary exploratory regression analyses of selected nutrition knowledge items and LDL and fasting glucose levels.

Scale Item	Metabolic Variables	$\bar{x}$ ± sd	β	95% CI for B	R^2^	F	t	*p*
4	LDL (mg/dL)	76.6 ± 27.3	−0.185	−0.017 to −0.003	0.034	8.042	−2.836	**0.005**
	Fasting glucose (mg/dL)	97.4 ± 14.2	−0.121	−0.027 to 0.001	0.015	3.408	−1.846	0.066
7	LDL (mg/dL)	76.6 ± 27.3	−0.201	−0.016 to −0.004	0.040	9.579	−3.095	**0.002**
	Fasting glucose (mg/dL)	97.4 ± 14.2	−0.072	−0.019 to 0.006	0.005	1.177	−1.085	0.279
13	LDL (mg/dL)	76.6 ± 27.3	−0.257	−0.020 to −0.007	0.066	16.146	−4.018	**<0.001**
	Fasting glucose (mg/dL)	97.4 ± 14.2	−0.064	−0.019 to 0.007	0.004	0.932	−0.965	0.335

β: Standardized regression coefficient; B: Unstandardized regression coefficient; 95% CI: 95% confidence interval for B.

**Table 6:** Comparison of selected nutrition knowledge item responses according to daily meal consumption groups.

Scale Item	Meal Consumption	*n*	$\bar{x}$ ± sd	F	*p*	Post Hoc (^1^ Tukey; ^2^ Games–Howell)	η^2^
4	1–2 meals (A)	117	2.50 ± 1.45	4.860	**0.009**	A-B ^1^, A-C ^1^	0.041
	3–4 meals (B)	101	2.01 ± 1.53				
	≥4 meals (C)	12	1.42 ± 1.51				
6	1–2 meals (A)	117	1.64 ± 1.22	3.412	**0.035**	A-B ^2^	0.029
	3–4 meals (B)	101	2.06 ± 1.32				
	≥4 meals (C)	12	2.25 ± 1.77				
8	1–2 meals (A)	117	2.50 ± 1.38	5.429	**0.005**	A-C ^1^	0.046
	3–4 meals (B)	101	2.10 ± 1.41				
	≥4 meals (C)	12	1.25 ± 1.55				

## Data Availability

The data used in this research can be obtained from the corresponding author upon reasonable request.

## Abbreviations

The following abbreviations are used in this manuscript:

HDL	High-density lipoprotein
LDL	Low-density lipoprotein
BMI	Body mass index
SPSS	Statistical Package for the Social Sciences
TL	Turkish lira
DNA	Deoxyribonucleic acid
